# War related sexual violence and it's medical and psychological consequences as seen in Kitgum, Northern Uganda: A cross-sectional study

**DOI:** 10.1186/1472-698X-10-28

**Published:** 2010-11-10

**Authors:** Eugene Kinyanda, Seggane Musisi, Christine Biryabarema, Isaac Ezati, Henry Oboke, Ruth Ojiambo-Ochieng, Juliet Were-Oguttu, Jonathan Levin, Heiner Grosskurth, James Walugembe

**Affiliations:** 1 MRC/UVRI Uganda Research Unit on AIDS, P.O. Box 49, Entebbe, Uganda; 2 Department of Psychiatry, Makerere University, P.O. Box 7072, Kampala, Uganda; 3 Mulago National Referral Hospital, P.O. Box 7051 Kampala, Uganda; 4 Isis-Women's International Cross Cultural Exchange (Isis-WICCE), P.O. Box 4934, Kampala, Uganda; 5 Butabika National Psychiatric Referral Hospital, P.O. Box 7017 Kampala, Uganda

## Abstract

**Background:**

Despite the recent adoption of the UN resolution 1820 (2008) which calls for the cessation of war related sexual violence against civilians in conflict zones, Africa continues to see some of the worst cases of war related sexual violence including the mass sexual abuse of entire rural communities particularly in the Great Lakes region. In addition to calling for a complete halt to this abuse, there is a need for the systematic study of the reproductive, surgical and psychological effects of war related sexual violence in the African socio-cultural setting.

This paper examines the specific long term health consequences of war related sexual violence among rural women living in two internally displaced person's camps in Kitgum district in war affected Northern Uganda who accessed the services of an Isis-Women's International Cross Cultural Exchange (Isis-WICCE) medical intervention.

**Methods:**

The study employed a purposive cross-sectional study design where 813 respondents were subjected to a structured interview as part of a screening procedure for an emergency medical intervention to identify respondents who required psychological, gynaecological and surgical treatment.

**Results:**

Over a quarter (28.6%) of the women (n = 573) reported having suffered at least one form of war related sexual violence. About three quarters of the respondents had 'at least one gynaecological complaint' (72.4%) and 'at least one surgical complaint' (75.6%), while 69.4% had significant psychological distress scores (scores greater than or equal to 6 on the WHO SRQ-20). The factors that were significantly associated with war related sexual violence were the age group of less than or equal to 44 years, being Catholic, having suffered other war related physical trauma, and having 'at least one gynaecological complaint'. The specific gynaecological complaints significantly associated with war related sexual violence were infertility, chronic lower abdominal pain, abnormal vaginal bleeding, and sexual dysfunction. In a multivariable analysis the age group of less than or equal to 44 years, being Catholic and having 'at least one gynaecological complaint' remained significantly associated with war related sexual violence.

**Conclusion:**

The results from this study demonstrate that war related sexual violence is independently associated with the later development of specific gynaecological complaints.

## Background

Africa continues to experience some of the worst cases of war related sexual violence with the situation in some parts of the continent such as Eastern Democratic Republic of Congo characterized by indiscriminate sexual violence [1,2]. Sexual violence in these situations is mainly perpetrated by armed groups who use it to humiliate, intimidate, and dominate women, girls, their male partners and entire communities [2]. This situation of mass sexual abuse of communities including the rape of children as young as 10 months has been described as "murderous madness" [1]. It is hoped that the adoption of the landmark UN resolution 1820 (2008) which, "demands the immediate and complete halt to acts of sexual violence against civilians in conflict zones" will focus world attention on this epidemic problem [3].

War related sexual violence for purposes of this study is considered to be equivalent to sexual torture which Lunde and Ortmann (1998) have described as presenting in four forms, namely: i) violence against the sexual organs; ii) physical sexual assault, i.e. sexual acts involving direct physical contact between victim and torturer, between victim and victim, between victim and an animal, or all three of the above; iii) mental sexual assault i.e. forced nakedness, sexual humiliations, sexual threats and witnessing others being sexually tortured; iv) a combination of the three above [4]. According to Lunde and Ortmann (1998) the important point about sexual violence is that the victim is subjected to involuntary sexual acts which because they are involuntary, are psychologically painful, whether or not they are also perpetrated with physical harm [4].

Long term health consequences of war related sexual violence include reproductive health problems, surgical problems and psychological health problems in the survivors. Reproductive health problems have been reported to include: pain in both external and internal genitalia; menstrual disturbances; urination and defecation problems; sexual problems such as sexual dysfunction; rectal and vaginal fistulae and traumatic genital injury; urinary tract infections; STDs including pelvic inflammatory disease, HIV/AIDS; disturbance of reproduction including infertility; and precancerous and/or cancerous cervical tumours [1,2,4-7]. Destruction of breast tissue may result in necrosis of the papillae so that a mother cannot breastfeed her infant, which is often the only available source of nutrition for babies in situations of war [4].

As surgical consequences, survivors of war related sexual violence may report uncharacteristic lower lumbar pain sometimes radiating to the pelvis or gluteal region, with many having difficulties in standing or sitting for long periods [4]. Psychological consequences of war related sexual violence include post traumatic stress disorder (PTSD), anxiety disorders including phobias, psychosomatic symptoms, psychogenic pain, conversion-dissociative disorder, major depressive disorder, self-injurious behaviour including suicidality, alcohol and substance abuse and altered self-image and view of the world [4,6,7].

However few systematic studies of the reproductive, surgical and psychological effects of war related sexual violence have been undertaken in the African socio-cultural setting.

### Setting of the study

Northern Uganda has suffered chronic warfare for the last 20 years, with a conflict waged between the central government army (the Uganda Peoples Defence Forces; UPDF) and a vicious rebel group, the Lord's Resistance Army. This conflict has led to the mass traumatisation of the population including the abuse of human rights, and the forced displacement of over 2 million people (80% of the population in the region) into internally displaced persons camps. Since 2006, the Sudan sponsored peace talks between the Uganda government and the Lord's Resistance Army have brought relative peace to the region with people gradually returning to their homes; as a result both government and the international community have begun focusing on the rehabilitation and resettlement of the war survivors. However often such rehabilitation and resettlement programs fail to address the mental health and reproductive health needs of war survivors; when these needs are addressed it is done in a very superficial manner.

This paper will examine the specific long term health consequences of war related sexual violence among women from two internally displaced persons camps in Kitgum district, in war affected Northern Uganda, with the aim of highlighting the burden of these problems.

## Methods

### Study Site

Study respondents were recruited in 2005 from 2 out of 21 internally displaced persons camps (IDPs) in Kitgum district (approximate population, 300,000) when war was still ongoing and the security situation in the region extremely precarious. The camps which were selected, Mucwini (population 22,438) and Padibe (population 35,006) were some of the biggest and worst affected by the war. This study was part of a medical intervention to offer emergency reproductive health care, psychological support and surgical services to women affected by the war [8]. The intervention was undertaken by Isis-WICCE (an international women's organization) working in collaboration with professional associations of gynaecologists, psychiatrists and surgeons of Uganda.

The medical intervention involved an initial screening stage where trained non-medical community interviewers resident in the two camps administered a structured screening questionnaire to identify respondents who had suffered war trauma and had psychological, gynaecological or surgical complications. Apart from documenting these problems through the screening interview, respondents who were found to require further medical evaluation and treatment were referred to the camp based health workers. Respondents who were found to require further evaluation and treatment including surgery were referred to the visiting team of specialist who were based at Kitgum district hospital. The study employed a purposive cross-sectional study design where respondents who wanted to access the services of the medical intervention were consecutively recruited and subjected to the screening interview.

### Research instruments

A structured questionnaire was used to screen the war survivors for war trauma and it's psychological and medical consequences. It included items on the following; a) Socio-demographics - change of district, sex, age, religion, marital status, highest educational attainment, and employment status, b) War trauma- derived from the commonly reported forms of war trauma in Uganda [9] grouped under i) loss of loved ones (5 items)- including death as a result of war of the spouse, children, parents or other close relatives, ii) war related sexual violence (11 items)- including single episode rape, gang rape, homosexual rape, sexual comforting and abduction with sex, iii) physical trauma (10 items)-including whether the respondent suffered beatings or being kicked, gunshot injury, burn injuries, being forced to carry heavy loads for long distances and severe tying of the hands behind the back (locally called *Kandoya*), iv) psychological trauma (6 items)- including whether the respondent witnessed someone being killed, ever being forced to sleep in the bush or swamps for days, weeks and whether ever abducted, c) Perpetrators of war trauma (9 items)- possible perpetrators of war trauma included the Lord's Resistance Army, government army (UPDF), police, and prisons officers, d) Psychological consequences - including psychological dependence on alcohol (alcoholism) assessed using the C.A.G.E [10], iii) psychological distress (assessed using the WHO Self Report Questionnaire-20 [11], 1994), iv) suicide attempt (whether had attempted suicide in a life-time and in the last 12 month), v) whether had homicidal ideas. e) Gynaecological problems[12,13] (13 items)- including leaking urine (vaginal fistula), leaking faeces (rectal fistula), vaginal and perineal tears, sexual dysfunction and abnormal vaginal discharge, f) Surgical problems[12,13] (9 items)- including chronic backache, broken limbs, disfigurement as a result of burns and swellings on limbs, g) HIV serological status, and h) personal risk assessment of contracting HIV over next few years-assessed as low, medium or high.

### Data entry and analysis

Questionnaires were reviewed every evening for errors and inconsistencies which were then brought to the attention of the interviewers. Data was entered using SPSS version 10.0. and analysed using STATA. War related sexual violence was the dependent variable. As part of data analysis, frequencies were generated, and unadjusted analyses undertaken using Chi square and Odds ratios as the test statistics. Factors found to be significantly associated with war related sexual violence in unadjusted analyses were entered into multiple logistic regression model to determine their independent contribution to war related sexual violence. For this multivariable analysis, a logistic regression model was fitted using a stepwise backward elimination procedure with a liberal significance level of 0.15 so that all potentially important variables would be included in the model. The model was then verified by forward selection.

### Ethical Considerations

Scientific and ethical clearance for the medical intervention and research was sought by Isis-WICCE from both the Ministry of Health of Uganda and the Uganda National Council of Science and Technology. Informed consent was obtained from the respondents after adequate explanation of the study procedures and both the immediate and anticipated benefits of the screening procedure/study. This manuscript was reviewed and cleared by the Uganda National Health Research Organisation (UNHRO).

## Results

### Sample characteristics

813 respondents were screened during this study of whom 573 (70.5%) were women or girls. The gender ratios displayed in this study probably reflect the manner in which the medical intervention was publicized in the camps- as the intervention mainly addressed women's health concerns. This analysis focused on the women and girls, with most of them (90.2%) displaced from within their mother district of Kitgum. The majority were over the age of 24 years (88.7%), mainly Christian (99.3%), married or ever have been married (82.3%), of no or little formal education (less than 8 years of formal education; 94.9%) and mainly employed as peasant farmers (87.6%).

### War related sexual violence and other forms of trauma

Over a quarter (28.6%) of the women and 6.7% of the men reported having suffered at least one form of war related sexual violence (Table 1). The most frequently reported forms of war related sexual violence among women included defilement (14.5%), abduction with sex (7.9%), attempted rape (4.9%), forced marriage (4.4%) and heterosexual rape-single episode (4.2%). Among the men the most frequently reported forms of war related sexual violence was homosexual rape (4.2%). Other commonly reported forms of war trauma experiences reported included, death of at least one loved one (67.7%), having suffered at least one physical trauma (52.0%), and having suffered at least one psychological trauma (86.7%).

**Table 1:** War related sexual violence among respondents from Kitgum, Northern Uganda (N = 813)

**Trauma event**^**#**^	Females (n = 573)		Males (n = 240)	
	n	%	n	%
Heterosexual rape- single episode	24	4.2	-	-
Heterosexual rape- gang rape	5	0.9	-	-
Homosexual rape	-	-	10	4.2
Attempted rape	28	4.9	2	0.8
Forced marriage	25	4.4	4	1.7
Sexual comforting	19	3.3	-	-
Defilement	83	14.5	-	-
Sex in exchange for gifts	11	1.9	2	0.8
Sex in exchange for food	9	1.6	1	0.4
Forced incest	10	1.7	4	1.7
Abduction with sex	45	7.9	4	1.7
**Suffered at least one form of sexual violence**	**164**	**28.6**	**16**	**6.7**

^#^Some respondents suffered more than one form of sex violence

### Perpetrators of war related violence among women

The main perpetrators of war trauma in general among women were the rebel Lord's Resistance Army (71.4%) and the government soldiers (23.4%), a similar pattern was also reflected for war related sexual violence where the main perpetrators were the rebel Lord's Resistance Army (80.5%) and the government soldiers (23.2%; Table 2),

**Table 2:** Perpetrators of war related violence among women from Kitgum, Northern Uganda (N = 573)

**Perpetrators**^**#**^	Perpetrated war related trauma in general (physical, psychological, sexual; N = 573)		Perpetrated war related sexual violence (n = 164)	
	n	%	n	%
Lord's Resistance Army (LRA)	409	71.4	132	80.5
Government Soldiers (UPDF)	134	23.4	38	23.2
Police	7	1.2	1	0.6
Local Defence Personnel (Para-military group)	48	8.4	15	9.1
Prison Officers	6	1.0	1	0.6
Previous Government soldiers	7	1.2	1	0.6

^# ^Some respondents suffered war related violence and sexual violence at the hands of more than one perpetrator group

Table 3, a comparison of the sexual violations committed by the rebel Lord's Resistance Army (LRA) and the government soldiers reveals that the main method of sexual trauma in both groups were defilement (LRA- 55.3%; government soldiers- 42.1%), abduction with sex (LRA- 27.3%; government soldiers- 36.8%) and attempted rape (LRA- 15.2%; government soldiers- 18.4%). There were however important differences between the two groups of perpetrators with government soldiers proportionally using more the method of sexual comforting (26.3%) than the LRA (10.6%) and the LRA proportionally using more the method of heterosexual rape- single episode (12.9%) than government soldiers (7.9%).

**Table 3:** A comparison of the sexual violations committed by the Lord's Resistance Army and the government soldiers among female respondents

**Sexual trauma event**^**#**^	Perpetrators of sexual violence	
	**Lord's Resistance Army (n = 132) **^**#**^	**Government soldiers (UPDF) (n = 38) **^**#**^
	n (%)	n (%)
Heterosexual rape- single episode	17 (12.9)	3 (7.9)
Heterosexual rape- gang rape	3 (2.3)	1 (2.6)
Attempted rape	20 (15.2)	7 (18.4)
Forced marriage	19 (14.4)	6 (15.8)
Sexual comforting	14 (10.6)	10 (26.3)
Defilement	73 (55.3)	16 (42.1)
Sex in exchange for gifts	9 (6.8)	4 (10.5)
Sex in exchange for food	8 (6.1)	2 (5.3)
Forced incest	6 (4.5)	2 (5.3)
Abduction with sex	36 (27.3)	14 (36.8)

^#^Some respondents suffered more than form of sexual violence

### Reproductive health problems and other morbidities

About three quarters (72.4%) of the respondents had 'at least one gynaecological complaint' with the most commonly reported complaints being chronic lower abdominal pain (52.2%), abnormal vaginal bleeding (27.4%), infertility (26.6%), genital sores (25.3%), and swellings in the abdomen (22.5%).

The respondents also had high levels of both psychological and surgical morbidity including alcoholism (18.7%), significant psychological distress (scores ≥6 on the WHO SRQ-20; 69.4%), attempted suicide life-time (15.8%), having at least one surgical complaint (75.6%) and known HIV positive status (4.8%) (although the majority of respondents (82.3%) did not know their HIV status.

### Correlates of war related sexual violence among women

Among women, the socio-demographic factors significantly associated with war related sexual violence were, age ≤ 44 years where 35.2% suffered war related sexual violence as compared to 19.3% in the ≥ 45 years age group (OR, 2.28; 95%CI, 1.52-3.40; p = < 0.001), and being Catholic where 32.3% suffered war related sexual violence as compared to 23.5% in the Protestants (OR, 1.55; 95%CI, 1.06-2.28; p = 0.04) (Table 4).

**Table 4:** Association between war related sexual violence and socio-demographic factors among women in Kitgum, Northern Uganda

	Experienced war related sexual violence (n = 164)		Did not experienced war related sexual violence (n = 409)				
Characteristics	n	%	n	%	**χ**^**2**^	Odds ratio (95% CI)	P-value
**Camp (573)**							
Mucwini	55	25.6	160	74.4	1.56	1	0.212
Padibe	109	30.4	249	69.6		1.27 (0.87-1.86)	
**Displaced from another district (n = 569)**							
Displaced	16	28.6	40	71.4	0.002	1	0.97
Not displaced	148	28.8	365	71.2		1.01(0.55-1.87)	
**Age group (n = 555)**							
≤ 44 years	117	35.2	215	64.8	16.56	2.28(1.52-3.40)	<0.001*
≥ 45 years	43	19.3	180	80.7		1	
**Religion (n = 571)**							
Protestant	51	23.5	166	76.5		1	
Catholics	113	32.3	237	67.7	6.67	1.55(1.06-2.28)	0.04*
Others (Muslims, traditional African faiths, etc)	0	0.0	4	100.0			
**Marital Status (n = 566)**							
Never married	30	30.0	70	70.0	0.11	1	0.74
Married/Ever been married	132	28.3	334	71.7		0.92(0.57-1.48)	
**Highest level of Education**							
No formal education	77	27.2	206	72.8	0.81	1	0.37
Primary level and above	87	30.6	197	69.4		1.18(0.82-1.70)	
**Employment status**							
Peasant farmer	147	29.3	355	70.7		1	
Fisherman	7	25.9	20	74.1	0.95	0.85(0.35-2.04)	0.62
Others	10	22.7	34	77.3		0.71(0.34-1.48)	

* Statistically significant association

Among women, the medical and psychosocial factors significantly associated with war related sexual violence were, suffered war related physical trauma (OR, 1.55; 95%CI, 1.07-2.24; p = 0.02), and having 'at least one gynaecological complaint' (OR, 2.86; 95%CI, 1.75-4.67; p = < 0.001) (Table 5).

**Table 5:** Relationship between war related sexual violence and psychosocial factors among women in Kitgum, Northern Uganda

	Experienced war related sexual violence (n = 164)		Did not experience war related sexual violence (n = 409)				
Characteristics	n	%	n	%	**χ**^**2**^	Odds ratio (95% CI)	P-value
**War related trauma**							
Suffered death of loved one as a result of war	106	27.3	282	72.7	1.00	0.82(0.56-1.21)	0.32
Suffered war related physical trauma	98	32.9	200	67.1	5.53	1.55(1.07-2.24)	0.02*
Suffered war related psychological trauma	149	30.0	348	70.0	3.39	1.74(0.96-3.16)	0.07
**Mental health problems**							
Alcoholism (CAGE positive)	33	30.8	74	69.2	0.32	1.14(0.72-1.80)	0.57
Attempted suicide (life-time)	31	35.6	56	64.4	1.90	1.40(0.87-2.27)	0.17
Significant psychological distress scores (SRQ scores ≥ 6)	112	29.2	271	70.8	0.04	1.04(0.70-1.55)	0.84
Feel like killing someone	25	39.1	39	60.9	3.67	1.69(0.98-2.89)	0.06
**Medical complaints**							
Having at least one gynecological complaint	130	34.4	248	65.6	18.54	2.86(1.75-4.67)	<0.001*
Have at least one surgical complaint	131	30.3	302	69.7	2.31	1.41(0.91-2.19)	0.13
**Personal risk assessment of contracting HIV over next few years**							
High	33	23.7	106	76.3		1	
Medium	47	28.1	120	71.9	5.32	1.25(0.75-2.11)	0.07
Low	75	34.9	140	65.1		1.72(1.06-2.78)	
**HIV status (self report)**							
Positive	9	33.3	18	66.7		1	
Negative	19	26.0	54	74.0	0.56	0.70(0.27-1.83)	0.76
Don't know	135	29.1	329	70.9		0.82(0.36-1.87)	

* Statistically significant association

The specific gynaecological complaints associated with war related sexual violence included infertility (OR, 2.16; 95%CI, 1.44-3.24; p = < 0.001), chronic lower abdominal pain (OR, 1.98; 95%CI, 1.35-2.91; p = < 0.001), abnormal vaginal bleeding (OR, 1.86; 95%CI, 1.24-2.79; p = 0.002) and sexual dysfunction (OR, 2.09; 95%CI, 1.27-3.45; p = 0.003) (Table 6).

**Table 6:** Relationship between reproductive health complaints to war related sexual violence among women in Kitgum, Northern Uganda

	Experienced war related sexual violence (n = 164)		Did not experienced war related sexual violence (n = 409)				
**Problems**^**#**^	n	%	n	%	**χ**^**2**^	Odds Ratio	P-value
Abnormal vaginal discharge	52	33.8	102	66.2	2.28	1.36(0.91-2.04)	0.13
Vaginal and perineal tear	31	36.5	54	63.5	2.58	1.49(0.91-2.42)	0.11
Leaking urine	23	30.3	53	69.7	0.07	1.07(0.63-1.82)	0.80
Leaking faeces	11	28.9	27	71.1	0.001	1.00(0.48-2.04)	0.97
Genital laxity/prolapse	17	40.5	25	59.5	2.87	1.74(0.91-3.32)	0.09
Infertility	59	41.5	83	58.5	14.23	2.16(1.44-3.24)	<0.001*
Chronic lower abdominal pain	100	35.7	180	64.3	12.41	1.98(1.35-2.91)	<0.001*
Abnormal vaginal bleeding	57	39.0	89	64.3	9.28	1.86(1.24-2.79)	0.002*
Swelling(s) in the abdomen	40	33.3	80	66.7	1.27	1.28(0.83-1.99)	0.26
Genital sores	47	35.1	87	64.9	2.74	1.42(0.94-2.16)	0.10
Unwanted pregnancy	17	31.5	37	68.5	0.14	1.12(0.61-2.06)	0.71
Sexual dysfunction	33	43.4	43	56.6	8.69	2.09(1.27-3.45)	0.003*
Having at least one gynecological complaint	130	34.4	248	65.6	18.54	2.86(1.75-4.67)	<0.001*

* Statistically significant association

### Multivariable Model

The logistic regression model computed showed that 2 domains of factors were independently associated with war related sexual violence, that is the predisposing factors-age of less than or equal to 44 years (AOR, 1.96; 95%CI, 1.23-3.13; p = 0.004) and being Catholic (AOR, 1.65; 95%CI, 1.07-2.54; p = 0.03) and the medical consequences of war related sexual violence-suffering from a gynaecological problem (AOR, 2.36; 95%CI, 1.39-4.00; p = 0.002) (Table 7).

**Table 7:** Logistical regression model for factors associated with war related sexual violence in female respondents

**Factors**^**1**^	Adjusted OR	95% CI	P-value
***Socio-economic factors***			
*Age group*			
≤ 44 years	1.96	(1.23-3.13)	0.004*
≥ 45 years	1		
*Religion*			
Protestant	1		
Catholic	1.65	(1.07-2.54)	0.03*
***Medical consequences***			
Having at least one gynaecological complaint	2.36	(1.39-4.00)	0.002*
Personal assessment of risk of contracting HIV/AIDS over next few years			
High	1		
Medium	1.05	(0.59-1.84)	0.88
Low	1.73	(1.02-2.94)	

^1^Pseudo R^2 ^0.07

## Discussion

This paper set out to examine the specific long term health consequences of war related sexual violence among women in war affected Northern Uganda.

The results of this study show that war related sexual violence was widespread during the 20 year conflict in Northern Uganda, in agreement with previous studies undertaken in the region [7,12,14]. These violations were not only committed by rebels of the Lord's Resistance Army, but also in the case of a quarter of survivors, by government soldiers. The range of sexual violations experienced by the women not only included those that were outright violent (rape, abduction with sex, forced incest), but also those that were coercive in nature (sex in exchange for gifts, defilement and sexual comforting) which took advantage of the women and girls increased vulnerability due to the war and it's associated mass poverty.

The women and girls most at risk for sexual violence were those aged 44 years or less, the reproductive age group. Although the association between belonging to the Catholic religion and suffering sexual violence could not readily be explained, membership to the Catholic church could have conferred increased vulnerability to war trauma inflicted by the rebels or the government for different reasons. In the case of rebels, since the LRA draws it's doctrinal roots from the Catholic church, they may have preferentially recruited from the Catholic church; in the case of the government soldiers, they may have targeted members of the Catholic church as sympathisers of the LRA. Another possible explanation for the association between being Catholic and suffering sexual violence could have been related to the misperception among both fighting groups that the Catholic women and girls were less likely to be infected by HIV/AIDS. In this study, physical trauma was significantly associated with war related sexual violence as reported elsewhere, not only because the two forms of trauma are usually concurrently administered together by the torturer, but also in many instances of sexual violence in war situations the survivors usually puts up physical resistance which is often overcome by the perpetrator using physical violence [2,4,6,7].

Experiencing war related sexual violence in the study was not associated with any of the parameters of psychological ill health, probably because the specific psychological effects of war related sexual violence were 'contaminated' by the psychological effects of physical and psychological trauma in this sample.

In this study war related sexual violence was independently associated with gynaecological complaints, which is in agreement with previous studies undertaken in this area [2,4,6 ]. This study has shown that not all gynaecological complaints were associated with war related sexual violence; it was those gynaecological complaints that were either symptoms of chronic pelvic inflammatory disease (i.e. chronic lower abdominal pain) or that were secondary complications of this (i.e. infertility, abnormal vaginal bleeding and sexual dysfunction) that were associated with war related sexual violence. Sexual dysfunction may have been associated with war related sexual violence for psychological reasons probably reflecting unresolved psychological trauma, where the sexual act triggers flashbacks of past sexual violations [6].

## Conclusions

In conclusion, this study has demonstrated that war related sexual violence was a common occurrence during the 20 year old war in Northern Uganda. It also demonstrated a strong association between war related sexual violence and the later development of specific gynaecological complaints. These results have the following implications for the observance of human rights in sub-Saharan Africa and for dealing with the medical and psychological health consequences of their abuse:

Firstly, governments particularly in Africa, should be urged and supported not only to ratify and domesticate the various international conventions against war trauma including war related sexual violence, they should be held accountable to make sure that these laws are upheld within their areas of jurisdiction.

Secondly, there is an urgent need for the institutionalization of human rights education in the various training curricula on the continent, including the training of members of the armed forces, police and prisons service.

Thirdly, the fact that war survivors in this study had high levels of gynaecological, psychological and surgical morbidity calls for the integration of medical and psychological treatment in all rehabilitation and resettlement programs for survivors of the many conflicts on the African continent. Lastly, medical and psychological treatment services for war survivors and human rights courts should be sensitive to the unique presentation of victims of war related sexual violence particularly the women and girls who not only may be suffering from severe physical and psychological pain, but may also have to deal with rejection and stigmatization by their families and communities and the consequent need for privacy to discuss these problems.

## Abbreviations

Isis-WICCE: Isis-Women's International Cross Cultural Exchange.

## Competing interests

The authors declare that they have no competing interests.

## Authors' contributions

Concept: EK, RO-O, JW-O, CB, HO; Data collection: EK, SM, CB, HO, JW, IE; Data analysis: EK, JL, HG; First draft: EK, RO-O, JW-O, SM, HG; Final revision: EK, RO-O, JW-O, CB, HO, SM, CB, IE, HG.

JW passed on in January 2009.

## Pre-publication history

The pre-publication history for this paper can be accessed here:

http://www.biomedcentral.com/1472-698X/10/28/prepub
